# Neutrophil gelatinase-associated lipocalin in dehydrated patients: a preliminary report

**DOI:** 10.1186/1756-0500-4-435

**Published:** 2011-10-22

**Authors:** Constantine N Antonopoulos, Alexandros Kalkanis, Georgios Georgakopoulos, Theodoros N Sergentanis, Dimitrios N Rigopoulos

**Affiliations:** 1 Department of Internal Medicine, 401 General Army Hospital, Athens, Greece

**Keywords:** dehydration, Neutrophil Gelatinase-Associated Lipocalin, Ngal, biomarker, acute kidney injury

## Abstract

**Background:**

Acute kidney injury has been recognized as a major contributor to end stage renal disease. Although neutrophil gelatinase-associated lipocalin (Ngal) has been reported as a promising biomarker for early detection of acute kidney injury, no study has yet examined its potential clinical impact in patients with normal renal function. The purpose of current study is to investigate possible difference in serum Ngal levels between dehydrated and control patients.

**Findings:**

A total of twelve patients presented with symptoms of mild dehydration defined by history of diarrheas or vomiting and orthostatic (postural) hypotension and an age and sex matched group of twelve control patients were included. The two groups of patients did not seem to differ in basic clinical and laboratory parameters. Serum Ngal was higher in dehydrated patients when compared to control group (Ngal = 129.4 ± 25.7 ng/mL vs 60.6 ± 0.4 ng/mL, p = 0.02). Ngal was not correlated with age, hemoglobin, white blood cell count, red blood cell count, urea or creatinine.

**Conclusions:**

The presence of elevated Ngal levels in dehydrated patients may suggest its role as a very sensitive biomarker in even minimal and "silent" prerenal kidney dysfunction

## Findings

Renal impairment may be the result of a variety of renal or systemic diseases and may lead to renal failure [1]. Although the gold standard of renal failure's diagnosis is serial measurements of serum creatinine (Cr) [2,3], this biomarker is of little clinical importance in very early stages of renal disease. A novel biomarker, neutrophil gelatinase-asssociated lipocalin (Ngal), has been promising in evidencing renal impairment, even when changes in serum Cr level are undetectable [1].

In order to explore the possible role of Ngal in subclinical renal dysfunction, such as renal hypoperfusion, we prospectively evaluated twelve patients aged>18 years with medical history suggesting mild dehydration, accompanied by orthostatic hypotension. A group of twelve apparently healthy individuals with no history of dehydration and no evidence of orthostatic hypotension were treated as controls. Ethical approval was obtained by the Ethics Committee of the "401 General Army Hospital" in Athens, Greece and all patients gave informed consent.

Dehydrated and controls were similar in age and gender (75% males in each group) and in terms of basic laboratory tests (Hemoglobin; Hb, White blood cells; Wbc, Red blood cells; Rbc, Urea; Ur and Cr). Although no laboratory differences were detected between the two groups, serum Ngal was higher in statistically significant terms in dehydrated patients, when compared to the controls (serum Ngal = 129.4 ± 25.7 ng/mL versus 60.6 ± 0.4 ng/mL, correspondingly, p = 0.02). Ngal was not significantly correlated with age, Hb, Wbc, Rbc, Ur or Cr (Table 1).

**Table 1:** Demographic characteristics, laboratory investigation (mean ± SE) and correlation of Ngal (ng/mL) (R, p value) in dehydrated and control patients

	Dehydrated (n = 12)		Control (n = 12)		
Comparison parameter	Mean ± SE	R (p value*)	Mean ± SE	R (p value*)	p value**
Ngal (ng/mL)	129.4 ± 25.7	-	60.6 ± 0.4	-	0.02

Age (yrs)	53.3 ± 6.3	0.40 (0.20)	53.6 ± 2.2	0.13 (0.68)	0.96
Hb (g/dL)	14.0 ± 0.4	-0.44 (0.15)	13.8 ± 0.5	0.27 (0.40)	0.63
Wbc (K/μL)	9.3 ± 0.7	-0.15 (0.65)	8.0 ± 0.8	-0.06 (0.85)	0.23
Rbc (M/μL)	5.0 ± 0.1	-0.41 (0.18)	4.9 ± 0.1	0.48 (0.12)	0.72
Urea (mg/dL)	68.7 ± 15.2	0.53 (0.08)	38.7 ± 4.4	0.13 (0.68)	0.06
Cr (mg/dL)	1.3 ± 0.1	0.12 (0.72)	1.0 ± 0.1	0.26 (0.86)	0.17

* p value for Pearson correlation coefficients between Ngal (ng/mL) and the other comparison parameters for patients and controls

** p value for independent samples T Test between dehydrated patients and controls for each comparison parameter

Dehydrated patients showed significantly higher serum Ngal levels compared to controls. It seems that dehydration may be involved in a pathophysiological pathway that causes renal hypoperfusion [4], decreases in Glomerular filtration rate (GFR) [2] and alone may produce changes in glomerular epithelial cells, comparable to those seen in the post-ischemic kidney [5]. During this period, injury to brush border of proximal tubule cells may be present and may lead to non-detectable minimal acute tubular necrosis [6]. Although in mild dehydration only minor and, probably, reversible changes in tubular epithelial cells are apparent, dehydration as a form of prerenal acute kidney injury may be represented by a tubular enzymuria and a concomitant increase in serum Ngal [2]. Thus, very sensitive and early detectable biomarkers, such as Ngal, can provide evidence and early insight into epithelial cell injury, insufficient to cause frank necrosis and thus undetectable with routine laboratory investigation [2]. This underlines the fact that Ngal may be a sensitive biomarker in even minimal and reversible acute kidney dysfunction, as seen in dehydrated patients.

Despite its novelty this preliminary report has some limitations. It is a single-center cross-sectional study with no follow-up data and may probably need to be validated in a large randomized controlled trial. Mean Ur in the controls, although not significantly different from cases, might suggest some volume contraction or might be confounded by age, sex, muscle mass and ethnicity. This report also lacks simultaneous examination of urinary Ngal and eventually, a creatinine clearance (CrCl) and GFR evaluation would rule out a possible "silent" renal impairment.

Our future research agenda points to increasing the dataset size, examining the role of urinary and serum Ngal, as well as other more elaborate indices of renal function in dehydration and evaluating their role in dehydrated patients that return to euhydrated status after proper treatment. Exploration of the underlying pathophysiological pathway of Ngal excretion in the dehydrated and hypoperfused kidney may also seem a very promising and tempting field of research.

## Abbreviations

(Cr): Creatinine; (Ngal): Neutrophil gelatinase-asssociated lipocalin; (Hb): Hemoglobin; (Wbc): White blood cell; (Rbc): Red blood cell; (Ur): Urea; (GFR): Glomerular filtration rate.

## Competing interests

The authors declare that they have no competing interests.

## Authors' contributions

CNA participated in the design of the study, performed the statistical analysis and drafted the manuscript. AK, GG, TNS and DNR participated in its design and coordination and helped to draft the manuscript. All authors read and approved the final manuscript.
